# Supplementary material for: Interaction of a Homologous Series of Amphiphiles with P-glycoprotein in a Membrane Environment—Contributions of Polar and Non-Polar Interactions
Source: Pharmaceutics. 2023 Jan 3;15(1):174. doi: 10.3390/pharmaceutics15010174 (PMC9862096; doi:10.3390/pharmaceutics15010174)
Supplement: Supplementary file 1 [file pharmaceutics-15-00174-s001.zip › pharmaceutics-2036048-supplementary.pdf]

Supplementary Information for

Interaction of a homologous series of amphiphiles with P-glycoprotein in a membrane environment – Contributions of polar and non-polar interactions

Maria João Moreno,[1,2*] Hugo A. L. Filipe,[1,3*] Susana V. P. Cunha,[1] Cristiana V. Ramos,[1] Patrícia A. T. Martins,[1] Biebele Abel,[4,5] Luís M. S. Loura,[1,2,6], and Suresh V. Ambudkar [4]

1 Coimbra Chemistry Center - Institute of Molecular Sciences (CQC-IMS), Department of Chemistry, University of Coimbra, 3004-535 Coimbra, Portugal

2 CNC - Center for Neuroscience and Cell Biology, University of Coimbra, 3004-535 Coimbra, Portugal

3 CPIRN-IPG - Center of Potential and Innovation of Natural Resources, Polytechnic of Guarda, 6300-559 Guarda, Portugal

4 Laboratory of Cell Biology, CCR, National Cancer Institute, NIH, Bethesda, MD 20892, USA

5 Henry M. Jackson Foundation for the Advancement of Military Medicine, Inc. and Uniformed Services University of the Health Science, Bethesda, MD 20817, USA

6 Faculty of Pharmacy, University of Coimbra, 3000-548 Coimbra, Portugal

∗ corresponding authors: mmoreno@ci.uc.pt; hlfilipe@ipg.pt

Contents page

S1 – Effect of the NBD amphiphiles and vanadate on the ATPase activity from native membranes 2

S2 – Effect of the NBD amphiphiles on the photoaffinity labeling of P-gp with [^{125}I] IAAP 5

S3 – Correction for the effect of scatter on the fluorescence intensity of NBD-Cn 6

S4 – Bilayer setup for Molecular Dynamics simulations 8

S5 – Umbrella sampling simulations for NBD-Cn in the water/complex asymmetric membrane 10

S6 – Umbrella sampling simulations for NBD-Cn in the membrane/P-gp systems 11

S7 – Dynamics of P-gp embedded in the membranes 13

S8 – Lipid distribution in the P-gp containing membranes 15

S9 – Details of the interaction of NBD-Cn molecules with the membrane and with P-gp 17

S10 – Calculation of the local concentration of the amphiphiles in the distinct environment 27

S11 – Additional information for the re-analysis of the ATPase activity assay considering the local concentrations and several binding sites in P-gp's binding pocket 28

S12 – Additional information for the re-analysis of the IAAP displacement assay considering the local concentrations and several binding sites in P-gp's binding pocket 29

S1 – Effect of the NBD amphiphiles and vanadate on the ATPase activity from native membranes

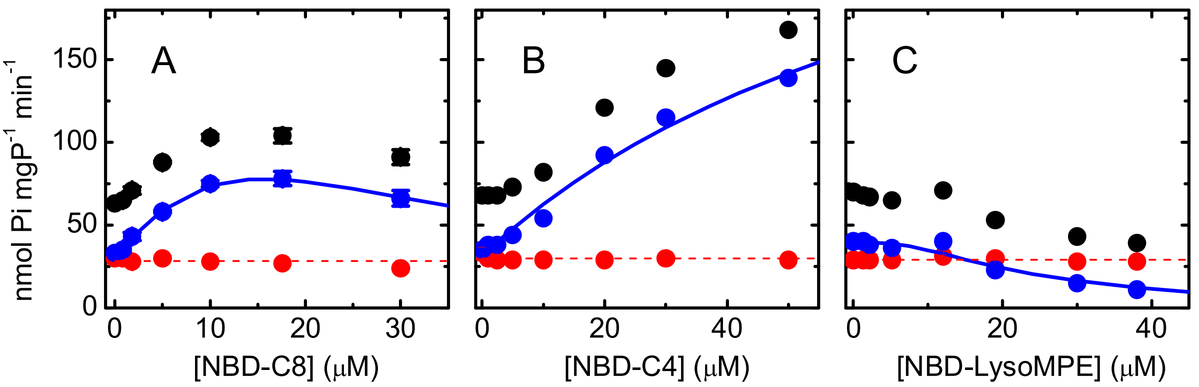

Figure S1 – Effect of the modulators NBD-C8 (plot A), NBD-C4 (plot B) and NBD-LysoMPE (plot C) on the rate of ATP hydrolysis of native membranes suspended in the ATPase assay buffer at a concentration of ca. 0.1 mg/mL. It is shown the total rate of ATP hydrolysis (●), the rate observed in the presence of 0.3 mM of vanadate (●), and the ATPase activity sensitive to vanadate which was calculated from the difference (●). The symbols represent the average of two measurements, with distinct aliquots from the same membrane preparation and modulator, vanadate and ATP added independently, the standard deviation is also shown (usually smaller than the symbol size). The lines are the average of the vanadate insensitive ATPase activity (- - -) and the best fit of the vanadate sensitive ATPase activity with equation S1 (—).

$$v = \frac{K_1 K_2 V_0 + K_2 V_1 [m] + V_2 [m]^2}{K_1 K_2 + K_2 [m] + [m]^2} \qquad \text{S1}$$

where [m] is the concentration of the modulator with respect to the total volume of the solution, V_0 is the basal ATPase activity, V_1 is the stimulatory activity, K_1 and K_2 being respectively the concentration of modulator leading to half stimulation and half inhibition, and V_2 the residual activity at infinite modulator concentration.

Figures S2 and S3 shows the sensitivity analysis of the different parameters in equation S2 on the value considered for V1 for the case of the effect of NBD-C8 on P-gp's ATPase activity. In Figure S2 it is considered that K_1 must be smaller than K_2, while in Figure S3 no relation is imposed between the two affinity constants.

Note that in Figure S2 the 75 % confidence interval includes the condition of equal affinity for the first and second modulator molecule ($K_1 = K_2$), and if no constraint is imposed between the two affinity constants (Figure S3) the affinity constants vary by more than an order of magnitude while the upper limit of the confidence interval is not even reached.

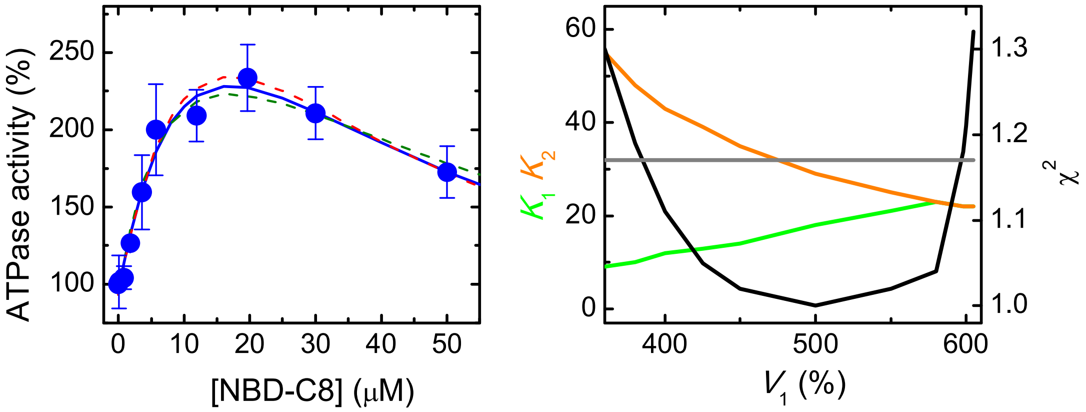

Figure S2 – Effect of NBD-C8 on the ATPase activity of m-Pgp in native membrane from High-Five insect cells. Left panel: the symbols are the average of 5 experiments (standard deviation shown as error bars). The continuous line is the best fit of equation S1, with V_1=497%, V_2 =0%, K_1=17 μM, and K_2 29 μM, and the dashed lines correspond to the best fit with the parameters at both extremes of the IC75%. Right panel: Dependence of the quality of the best fit of equation S2 (χ^2 —), and that of the parameters (K_1 — , and K_2 —) on the value assumed for the maximal activation (V_1). The threshold for a 75 % confidence interval is also shown (—).

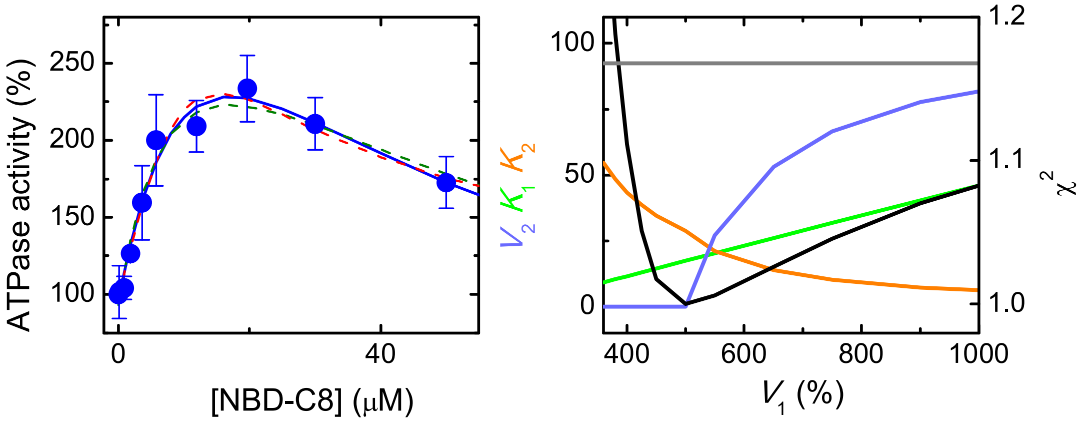

Figure S3 – Effect of NBD-C8 on the ATPase activity of m-Pgp in native membrane from High-Five insect cells. Left panel: the symbols are the average of 5 experiments (standard deviation shown as error bars). The continuous line is the best fit of equation S1, with V_1=497%, V_2 =0%, K_1=17 μM, and K_2 29 μM, , and the dashed lines correspond to the best fit with the parameters at both extremes of the IC75%. Right panel: Dependence of the quality of the best fit of equation S2 (χ^2 —), and that of the parameters (V_2 —, K_1 —, and K_2 —) on the value assumed for the maximal activation (V_1). The threshold for a 75 % confidence interval is also shown (—).

Table S1 – Parameters obtained from the best fit of equation S1 to the results obtained for the effect of the NBD amphiphiles on P-gp's the ATPase activity. The confidence intervals at 75% are also shown.

Parameter	NBD-C4	NBD-C8	NBD-LysoMPE
K_1 (μM)	**84** [59, 133]	**17** [10, 22] [a]<br>**17** [10, 160] [b]	**13** [2, 25] [a]<br>≥ **5** [5, ∞ [b]
K_2 (μM)	>> *60*	**29** [22, 48] [a]<br>**29** [1, 48] [b]	**13** [18, 25] [a]<br>≤ **15** [0, 15] [b]
V_1 (%)	**734** [604, 970]	**494** [380, 598] [a]<br>**494** [380, 3000] [b]	**106** [50, 120] [a]<br>**102** [100, 126] [b]
V_2 (%)	**0**	**0** [a]<br>**0** [0, 103] [b]	**0**<br>**0**

[a] imposing the constraint $K_1 \le K_2$; [b] no constraint between K_1 and K_2.

S2 – Effect of the NBD amphiphiles on the photoaffinity labeling of P-gp with [^{125}I] IAAP

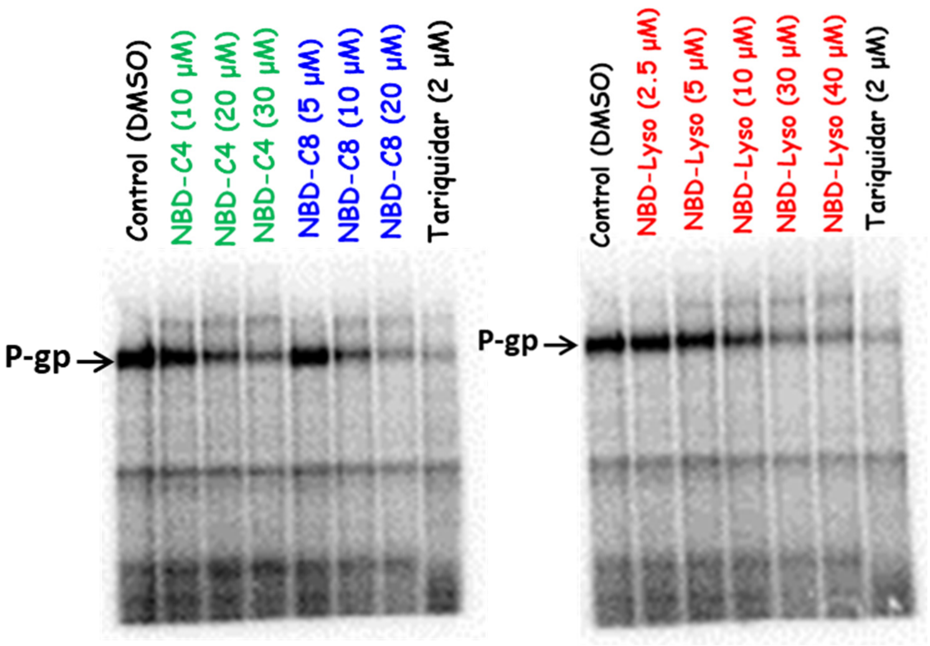

Figure S4 – Radioactivity incorporated into the P-gp band after incubation of native membranes with [^{125}I]IAAP and different concentrations of the NBD amphiphiles or 2 μM Tariquidar as indicated in the figure. The samples where photocross-linked with 366 nm UV light for 10 min at room temperature followed by SDS/PAGE electrophoresis and radioactivity quantification using the Storm 860 PhosphorImager system (Molecular Dynamics) and the software IMAGEQUANT.

$$\% \text{ inhibition} = 100 \times \frac{1}{1 + \frac{K_I}{[I]}\left(1 + \frac{[IAAP]}{K_M}\right)} \qquad S2$$

where K_M is the dissociation constant for IAAP, and K_I and [I] are the dissociation constant and the concentration of the competitive inhibitor (the NBD amphiphiles).

S3 – Partition of the NBD amphiphiles to lipid bilayers and native membranes

The fluorescence increase that accompanies the association of the NBD amphiphiles with the model membranes was well defined by a simple partition, equation S3.

$$\mathrm{If}_{540\mathrm{nm}} = \frac{\mathrm{If}^{\mathrm{W}} + \mathrm{If}^{\mathrm{M}} K_{\mathrm{P}}^{\mathrm{Lb}} \overline{V_{\mathrm{L}}} [L]}{1 + K_{\mathrm{P}}^{\mathrm{Lb}} \overline{V_{\mathrm{L}}} [L]} \qquad \text{S3,}$$

where If^{W} and If^{M} are respectively the fluorescence intensity when all fluorescent amphiphile is in the aqueous phase and associated with the membranes, $K_{\mathrm{P}}^{\mathrm{Lb}}$ is the partition coefficient between the aqueous and the lipid bilayer, $\overline{V_{\mathrm{L}}}$ is the molar volume of the lipid when in the lipid bilayer, and [*L*] is the lipid concentration.

To characterize the association of the NBD amphiphiles with the native membranes the whole membrane volume was considered, including the lipid bilayer and the membrane proteins. For the case of the less lipophilic amphiphiles NBD-C4 and NBD-C6, a significant scatter from the membranes was observed at the concentrations required for efficient partitioning. This is due both to the larger size of the vesicles (ca 1 µm diameter, [1]) and to the high refractive index of the native membranes. The magnitude of the scatter effect was quantified through the best fit of the fluorescence intensity of very lipophilic amphiphile NBD-C8, which shows efficient partition to the membranes at concentrations where scatter is not significant. The modified equation is:

$$\mathrm{If}_{540\mathrm{nm}} = \left(1 + s[M]\right) \frac{\mathrm{If}^{\mathrm{W}} + \mathrm{If}^{\mathrm{M}} K_{\mathrm{P}}^{\mathrm{W}\to\mathrm{M}} \left(\overline{V_{\mathrm{L}}}[L] + \overline{V_{\mathrm{P}}}[P]\right)}{1 + K_{\mathrm{P}}^{\mathrm{W}\to\mathrm{M}} \left(\overline{V_{\mathrm{L}}}[L] + \overline{V_{\mathrm{P}}}[P]\right)} \qquad \text{S4,}$$

where *s* is the scatter correction factor for a given concentration of native membranes, If^{W} and If^{M} are respectively the fluorescence intensity when all fluorescent amphiphile is in the aqueous phase and associated with the membranes, $K_{\mathrm{P}}^{\mathrm{W}\to\mathrm{M}}$ is the partition coefficient between the aqueous phase and the native membrane, $\overline{V_{\mathrm{L}}}$ is the molar volume of the lipid when in the membrane and [L] the molar concentration of the membrane lipids, $\overline{V_{\mathrm{P}}}$ is the volume occupied by 1 g of membrane protein (assuming a density of 1.2 g/mL) and [P] is the protein concentration in units of mass *per* volume.

Typical results obtained for the variation of the fluorescence intensity at 540 nm of the NBD-Cn fluorophores equilibrated with increasing concentrations of native membranes, are shown in figure S3. The fluorescence intensity was measured in black 96 well plates with the plate reader

SpectraMax iD5. The NBD-Cn concentrations were 0.5 μM for NBD-C8, 1 μM for NBD-C6, and 2 μM for NBD-C4, and the total volume *per* well was 150 μL.

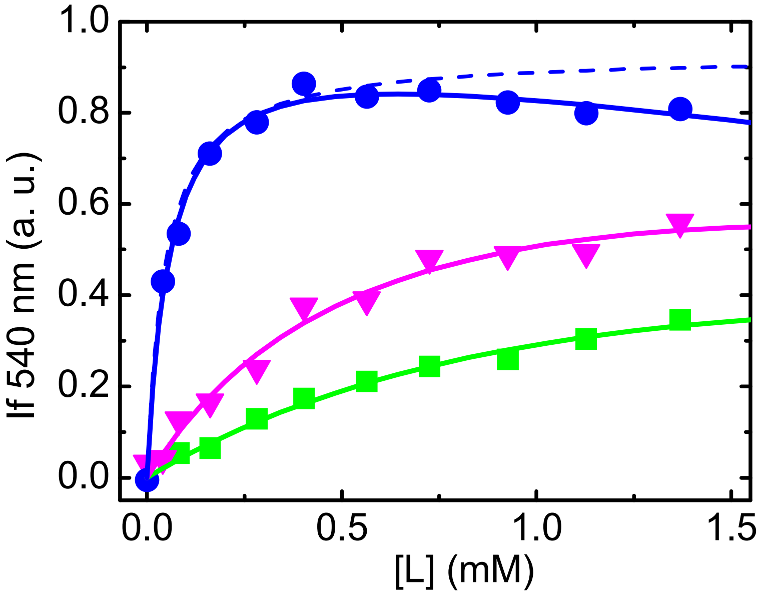

Figure S5 – Effect of scatter from the membranes, on the fluorescence intensity of NBD-Cn. For NBD-C8 (●), NBD-C6 (▼), and NBD-C4 (■), when measured by the plate reader SpectraMax iD5 in black 96 well plates with 150 μL *per* well. The fluorescence intensity was normalized by subtracting the signal in the absence of membranes (If^W) and dividing by the signal at complete association with the membrane (If^M). The lines correspond to the best fit of equation (S1).

At concentrations of native membranes above 0.5 mM phospholipid (corresponding to ≅ 0.7 mg/ml protein), most NBD-C8 is associated with the native membranes and its fluorescence intensity should remain essentially unchanged (dashed line in Figure S3). The decrease observed is due to artifacts introduced by the efficient light scatter from those samples (OD ≥ 0.6 at the excitation wavelength, 460 nm) which deviates the fluorescence signal from the detector. In conventional fluorimeters with 90 ° detection, this effect becomes significant at OD ≥ 0.1 and is so strong that makes the experiment unfeasible. However, in the reading setup of the plate reader SpectraMax iD5 most fluorescence intensity reaches the detector even in the presence of intense scatter. It is therefore possible to correct for the small effect observed at high scatter intensity.

The partition coefficient obtained for NBD-C8 (2.1×10^4 in the experiment shown in Figure S3) was in excellent agreement with the results obtained for small membrane concentrations (low scatter) in a conventional fluorimeter. The scatter correction factor obtained was included in the best fit of equation S4 to the results obtained for NBD-C6 and NBD-C4.

S4 – Bilayer setup for Molecular Dynamics simulations

Table S2 – Composition of the bilayer systems used in the MD simulations.

	Asymmetric membrane without P-gp		Asymmetric membrane with P-gp		POPC membrane with P-gp	
Lipid	Inner leaflet	Outer leaflet	Inner leaflet	Outer leaflet	Inner leaflet	Outer leaflet
POPC	91 (19)[a]	188 (38)	84 (19)	178 (38)	257 (100)	265 (100)
POPE	135 (27)	29 (6)	124 (27)	27 (6)		
SM	48 (10)	101 (20)	44 (10)	96 (20)		
POPS	58 (11)		53 (11)			
Cholesterol	150 (31)	164 (34)	138 (31)	155 (34)		
Total lipids	482	482	443	456	257	265
Water beads	27932		21628		12901	
Ion beads	617		480		284	
Size [b]	20 × 20 × 10		15 × 15 × 20		13.5 × 13.5 × 20	

[a] – number of lipid molecules (mol % with respect to all lipid molecules in that leaflet).

[b] – Size of the simulation box in nanometers; side × side × height.

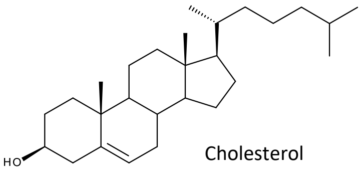

Cholesterol

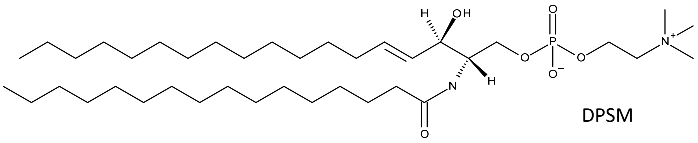

DPSM

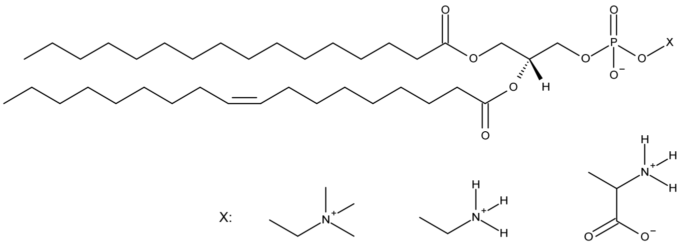

POPC POPE POPS

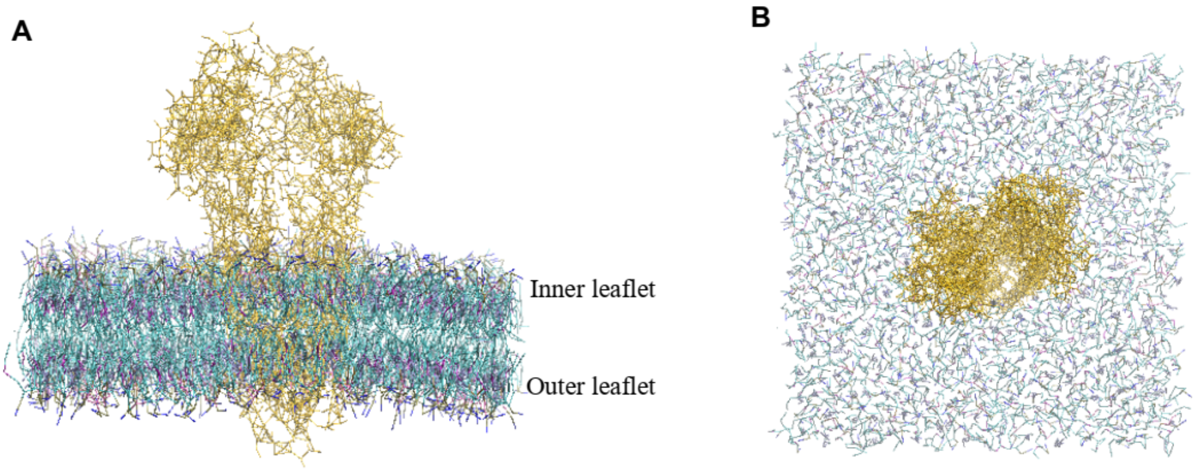

Figure S6. Snapshot of the complex membrane system containing one molecule of P-gp: A) side view and B) upper view of the Nucleotide Binding domains (NB-domains). The P-gp is shown in yellow, with the NB-domains on top. Water surrounding the membrane and P-gp is not shown.

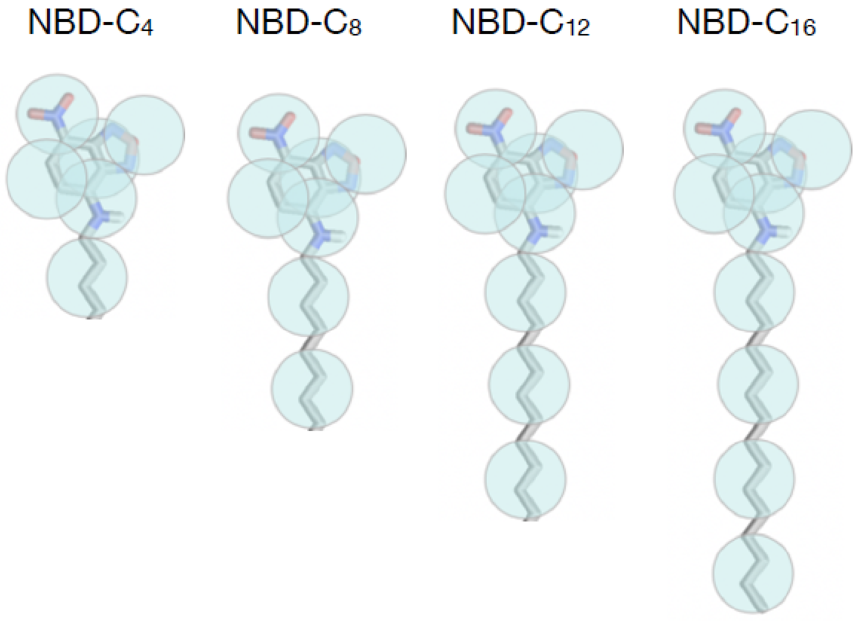

Figure S7. Coarse grain (CG) mapping of the NBD-Cn.

S5 – Umbrella sampling simulations for NBD-Cn in the water/complex asymmetric membrane

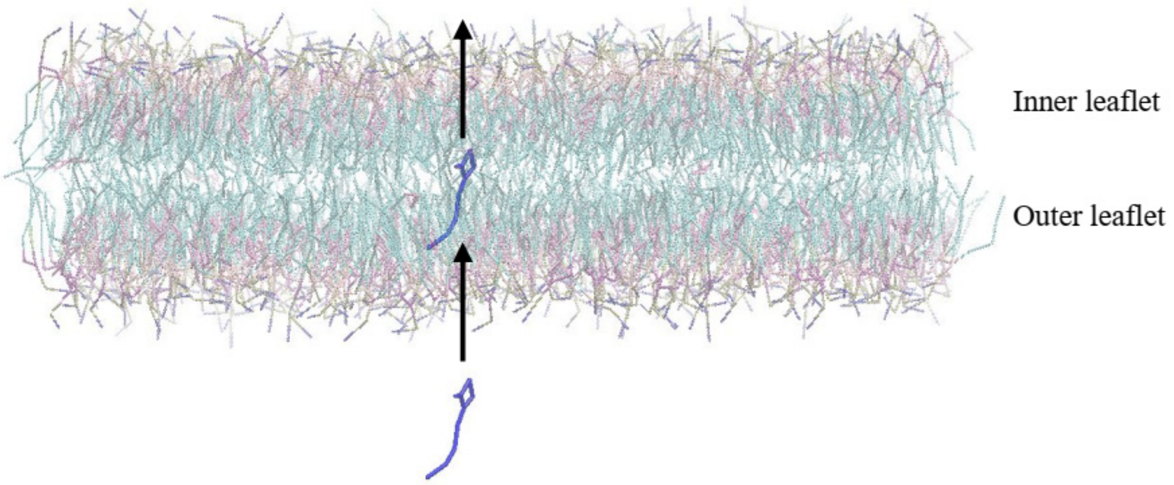

Figure S8. Initial positions and direction of pulling NBD-C16 (in dark blue) in the membrane/water system. Two molecules were added in each system. One was placed in the water and another with the NBD group in the center of the bilayer, 4 nm apart. Water above and below the membrane is not shown.

S6 – Umbrella sampling simulations for NBD-Cn in the membrane/P-gp systems

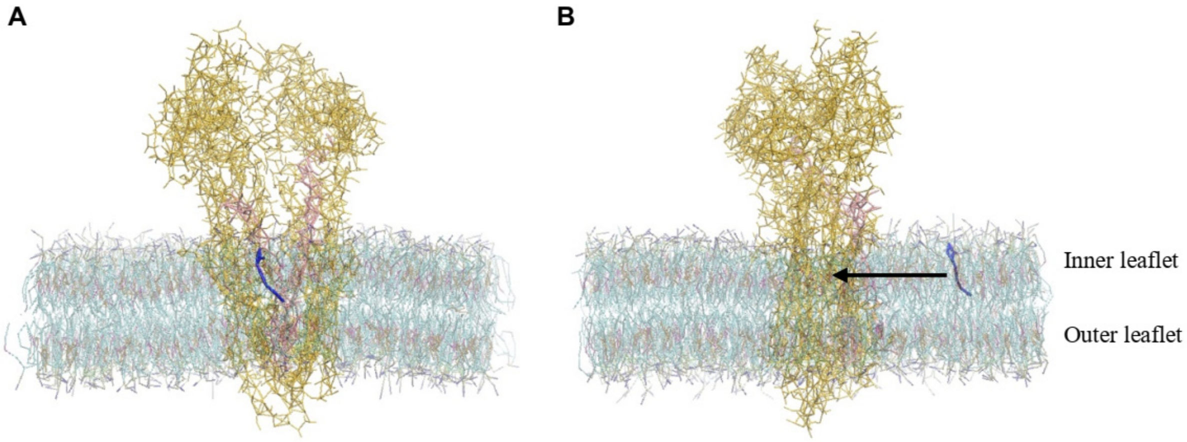

Figure S9. Initial position and direction of pulling of NBD-C16 from the membrane to the P-gp: A) front view and B) side view. The NBD-C16 molecule was placed at d=5 nm (distance in the xy plane to P-gp TM COM. The P-gp is shown in yellow, with the TM4 and TM6 gate in pink, and NBD-C16 is shown in dark blue. Water above and below the membrane is not shown.

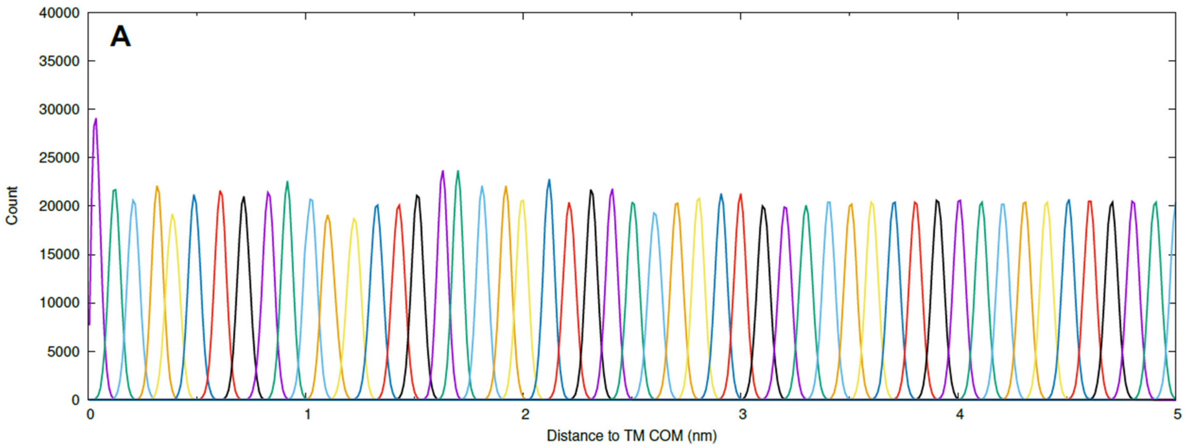

Figure S10. Sampling histograms with the probability distribution of the molecules in the umbrella windows along the reaction coordinate, for NBD-C8 in the P-gp/membrane system.

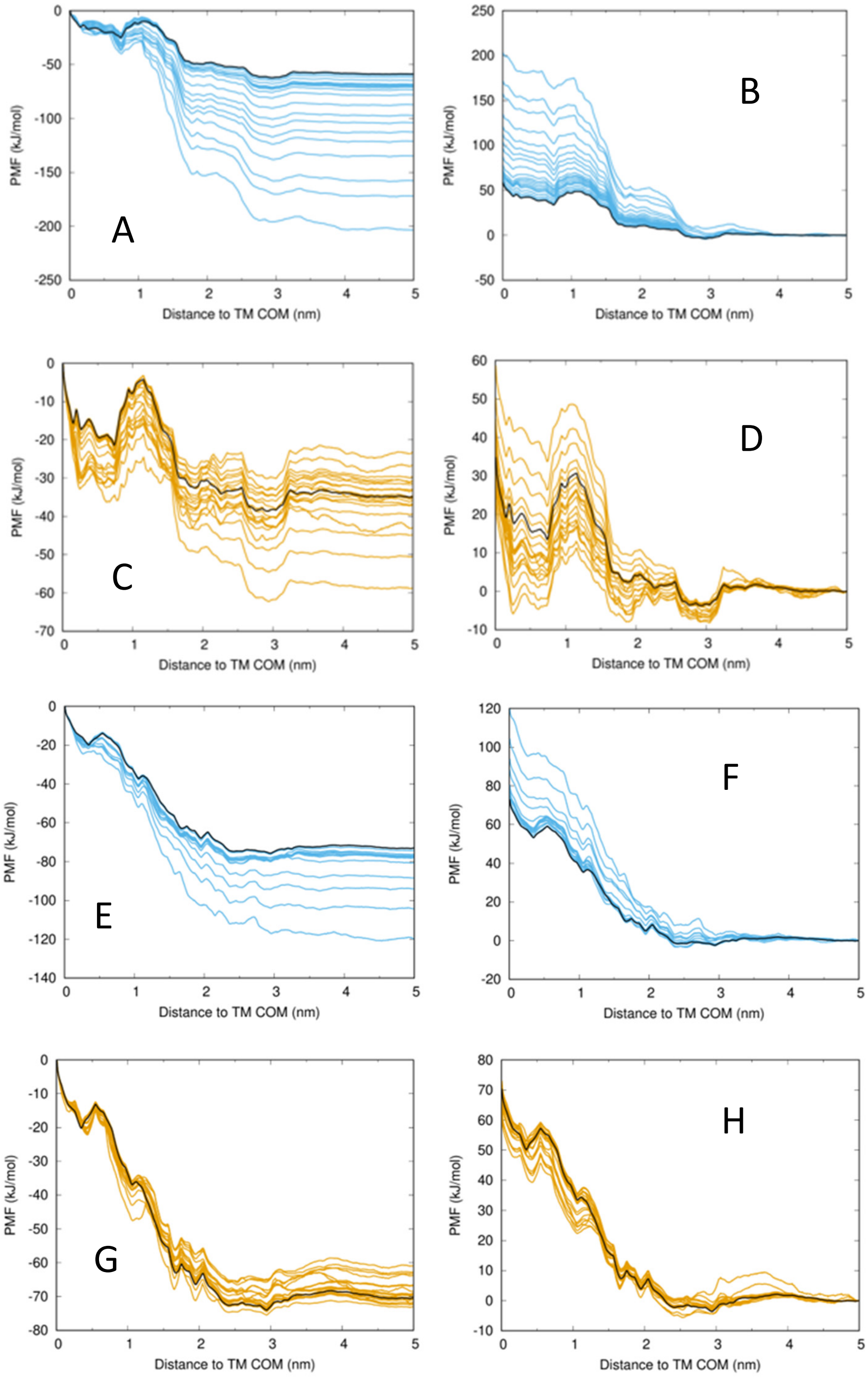

Figure S11. Convergence of the potential of mean force (PMF) profiles for the transfer of NBD-C4 (A to D) and NBD-C8 (E to H) from the membrane to P-gp in the plasma membrane model. Plots A and B (E and F) show profiles for increasing simulation times by a 10 ns interval, until 200 ns (in black); Plots C and D (G and H) show profiles obtained by disregarding initial simulation times by a 10 ns interval, until 200 ns. The profile for 50 ns as equilibration time is shown in black. The energy reference position is at 0 nm (plots A, C, E and G), or at 5 nm (plots B, D, F, and H) from the TM COM.

S7 –Dynamics of P-gp embedded in the membranes

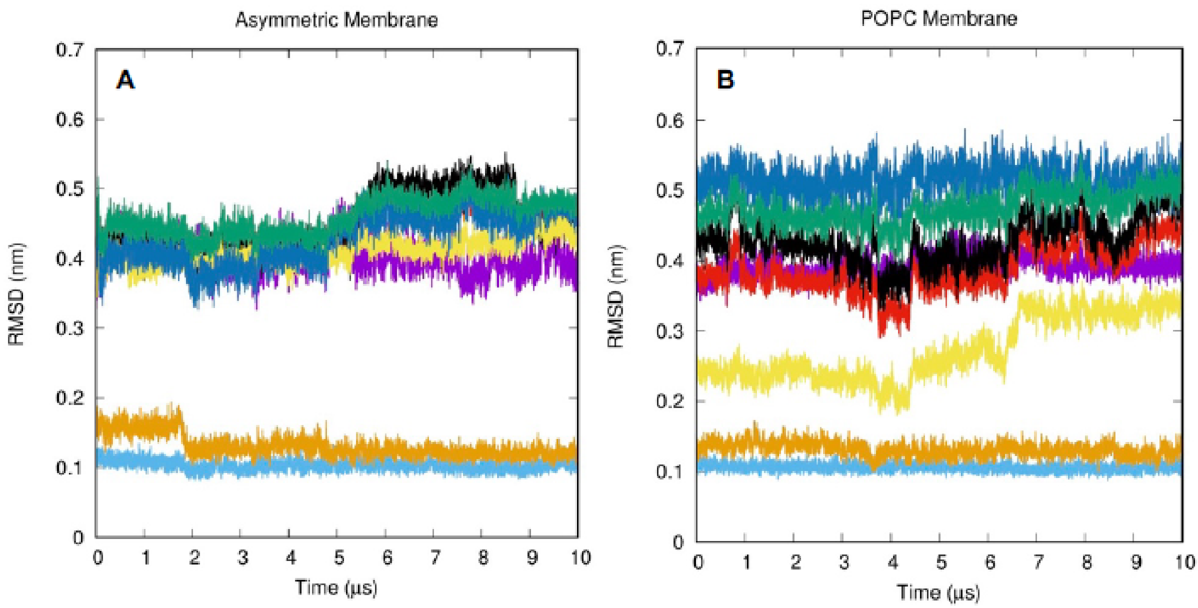

Figure S12. Root-Mean-Square Deviation (RMSD) of the backbone beads of diferent P-gp domains during the 10 μs simulation for the protein inserted in the asymmetric membrane (plot A) and in the POPC membrane (Plot B). RMSD calculated for the whole protein (—), the TM-domains (—), TM-domain 1 (—), TM-domain 2 (—), NB-domains (—), NB-domain 1 (—), NB-domain 2 (—), and linker (—).

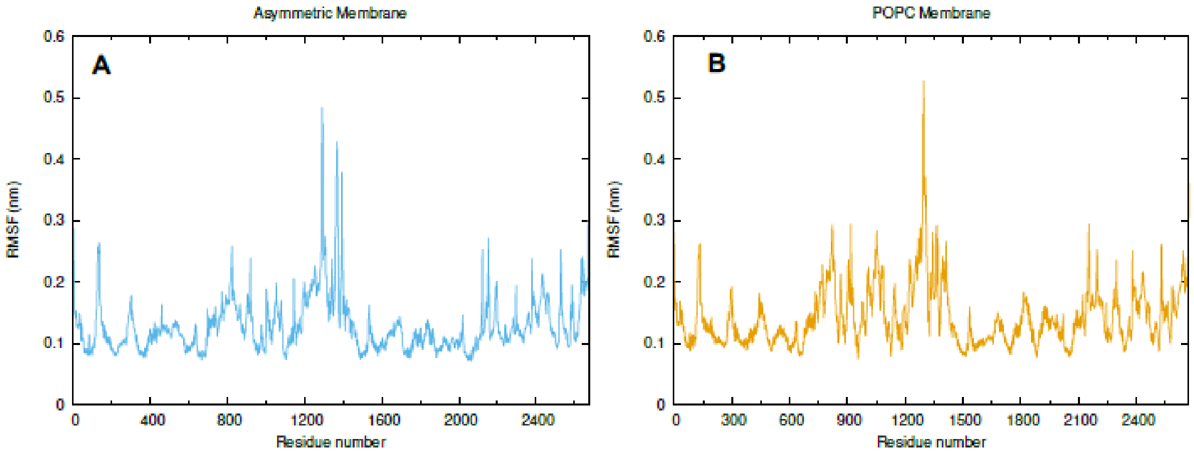

Figure S13. Root-Mean-Square Fluctuation (RMSF) of the backbone beads of P-gp residues during the 10 μs simulation for the protein inserted in the asymmetric membrane (plot A) and in the POPC membrane (Plot B).

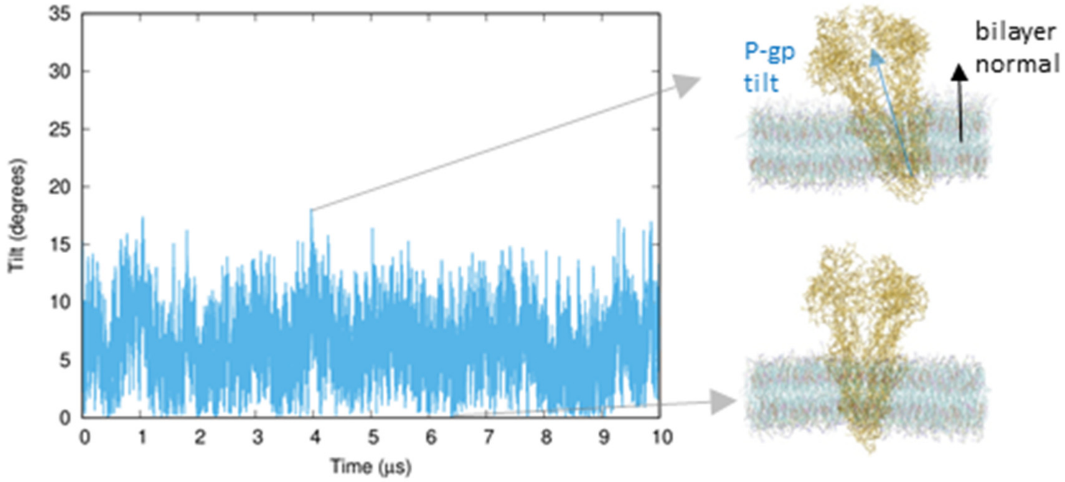

Figure S14. Time evolution of the tilt angle observed for P-gp inserted in the asymmetric lipid bilayers. The snapshot with the maximal (18.11 ^{o}) and minimal (0.02 ^{o}) tilt observed is also shown, with P-gp in front view shown in yellow. Water was omitted for clarity.

S8 – Lipid distribution in the P-gp containing membranes

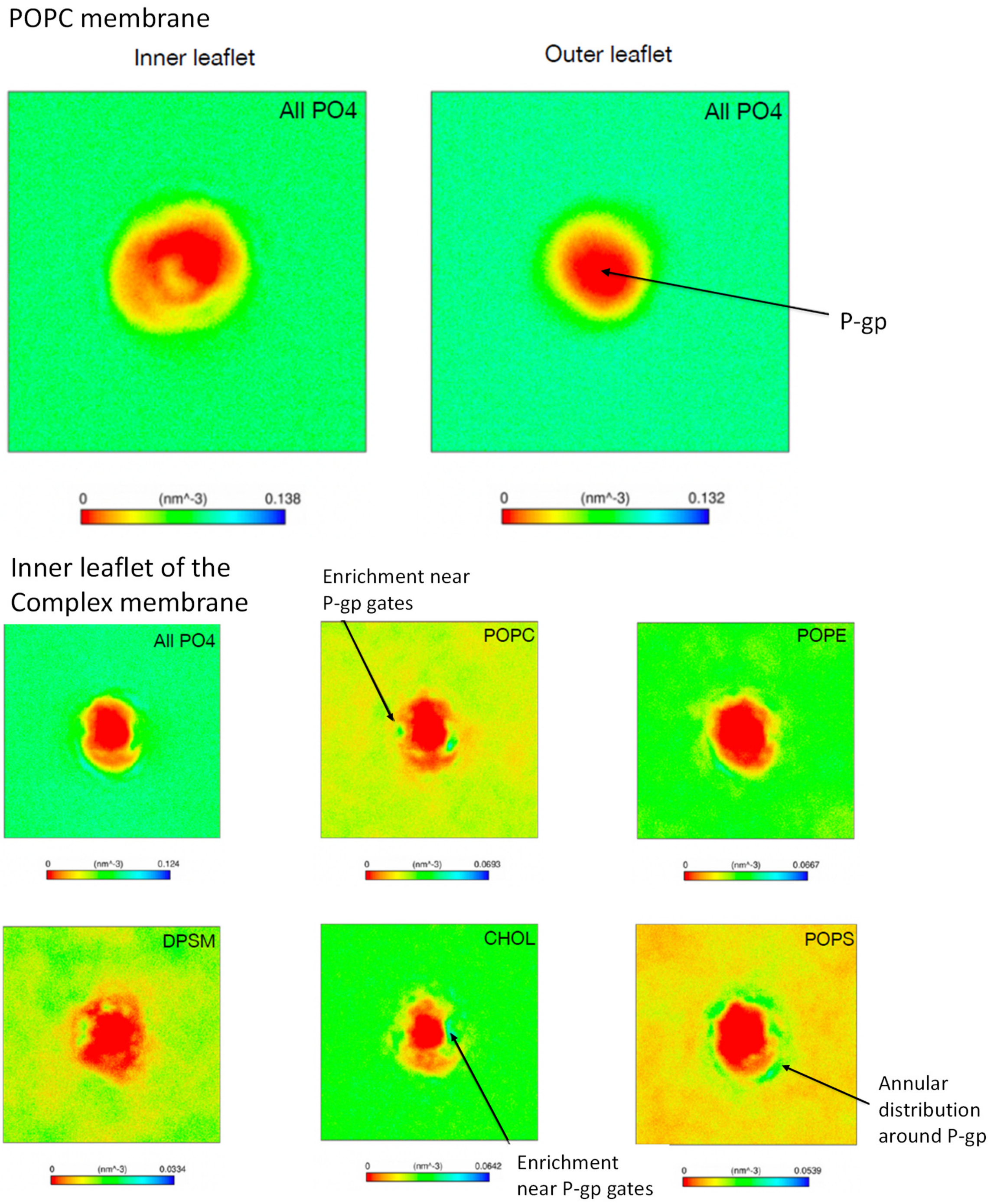

Figure S15. Lipid density maps averaged over the 10 µs simulation for P-gp embedded in the POPC membrane, and in the inner leaflet of the complex membrane. The density values are indicated by the color scale bars. The region in red corresponds to the space occupied by P-gp.

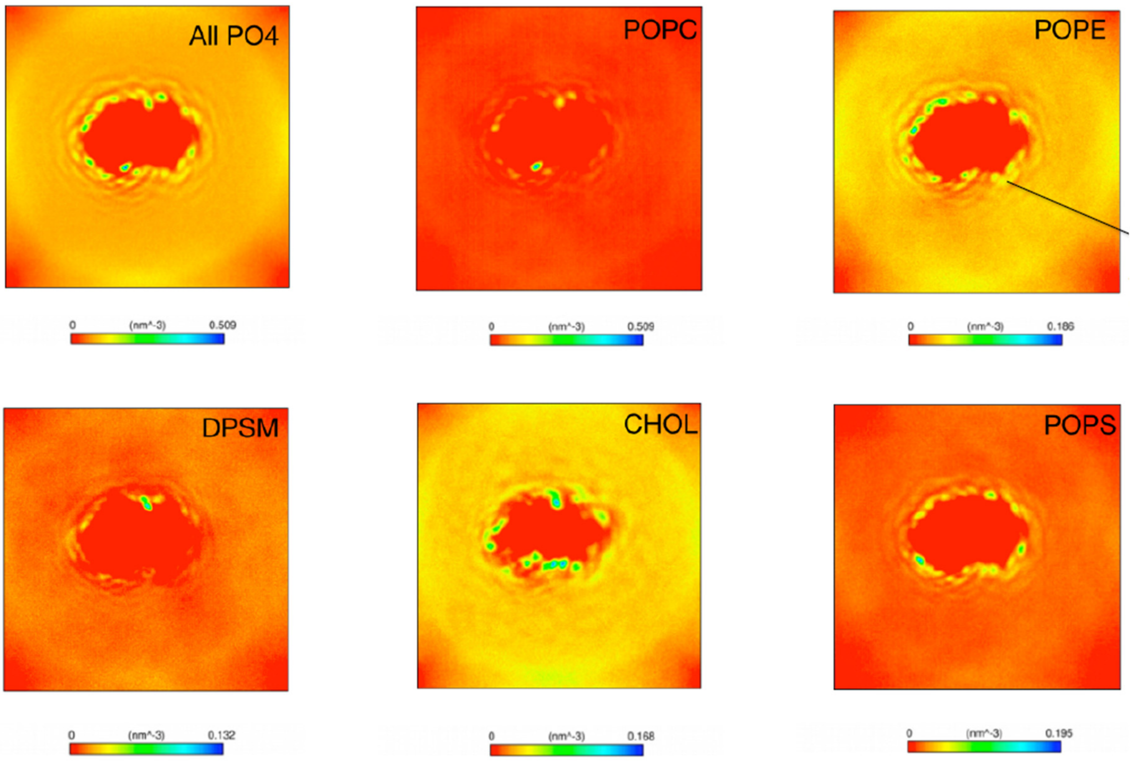

Figure S16. Lipid density maps for the lipids in the inner leaflet of the P-gp containing complex membrane. The lipid density was calculated taking into account for rotational orientation changes of P-gp during the 10 μs simulation.

S9 – Detailed characterization of the interaction of NBD-Cn molecules with the membrane and with P-gp

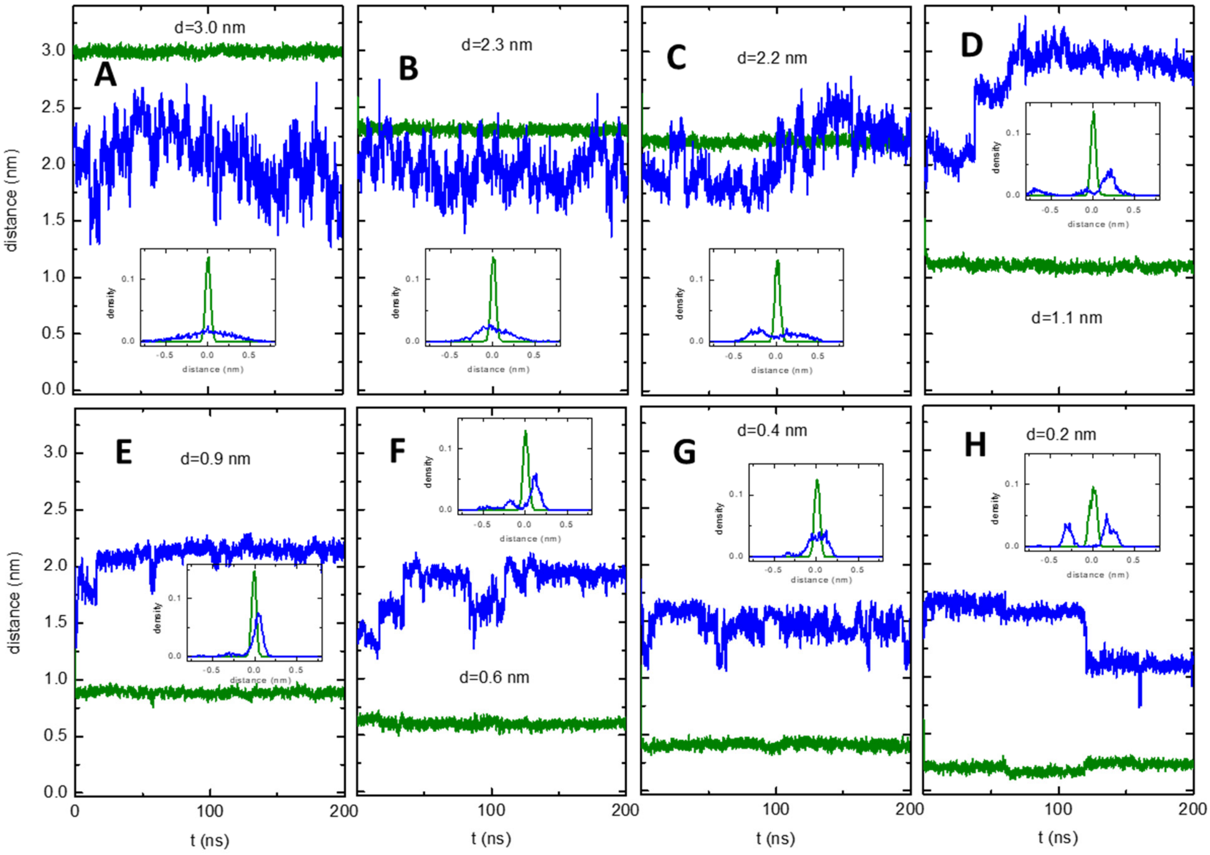

Figure S17 – Transverse location of the NBD group of NBD–C4 (*z*, —) and radial distance to the TM COM of P–gp (*d*, —) as a function of the simulation time in the windows corresponding to a restrained distance to the TM COM of P–gp (*d* = 3 nm (**A**), in the lipid bilayer; *d* = 2.2 and 2.3 nm (**B**,**C**), approaching the P–gp outer surface; *d* = 1.1 nm and 0.9 nm (**D**,**E**), near P–gp's entry gate; and *d* = 0.6, 0.4 and 0.2 nm (**F**,**G**,**H**), inside the P–gp binding pocket). The insets show the density of the NBD group around its average position in each window relative to the transverse (z, —) and to the constrained radial (*d*, —) distance.

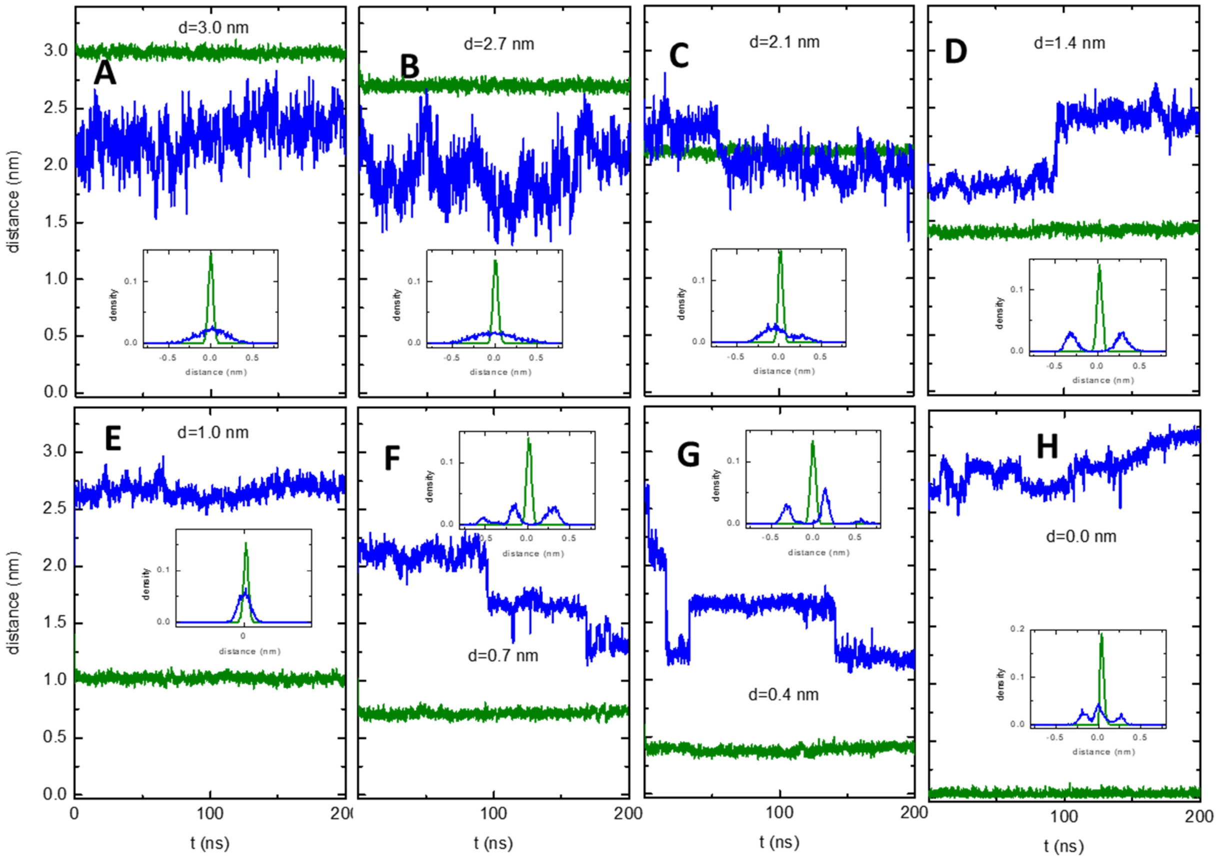

Figure S18 – Transverse location of the NBD group of NBD–C8 (*z*, —) and radial distance to the TM COM of P–gp (*d*, —) as a function of the simulation time in the windows corresponding to a restrained distance to the TM COM of P–gp (*d* = 3 nm and 2.7 nm (**A**,**B**), in the lipid bilayer; *d* = 2.1 nm (**C**), approaching the P–gp outer surface; *d* = 1.4 nm and 1.0 nm (**D**,**E**), near P–gp's entry gate; and *d* = 0.7, 0.4 and 0.9 nm (**F**,**G**,**H**), inside the P–gp binding pocket). The insets show the density of the NBD group around its average position in each window relative to the transverse (z, —) and to the constrained radial (*d*, —) distance.

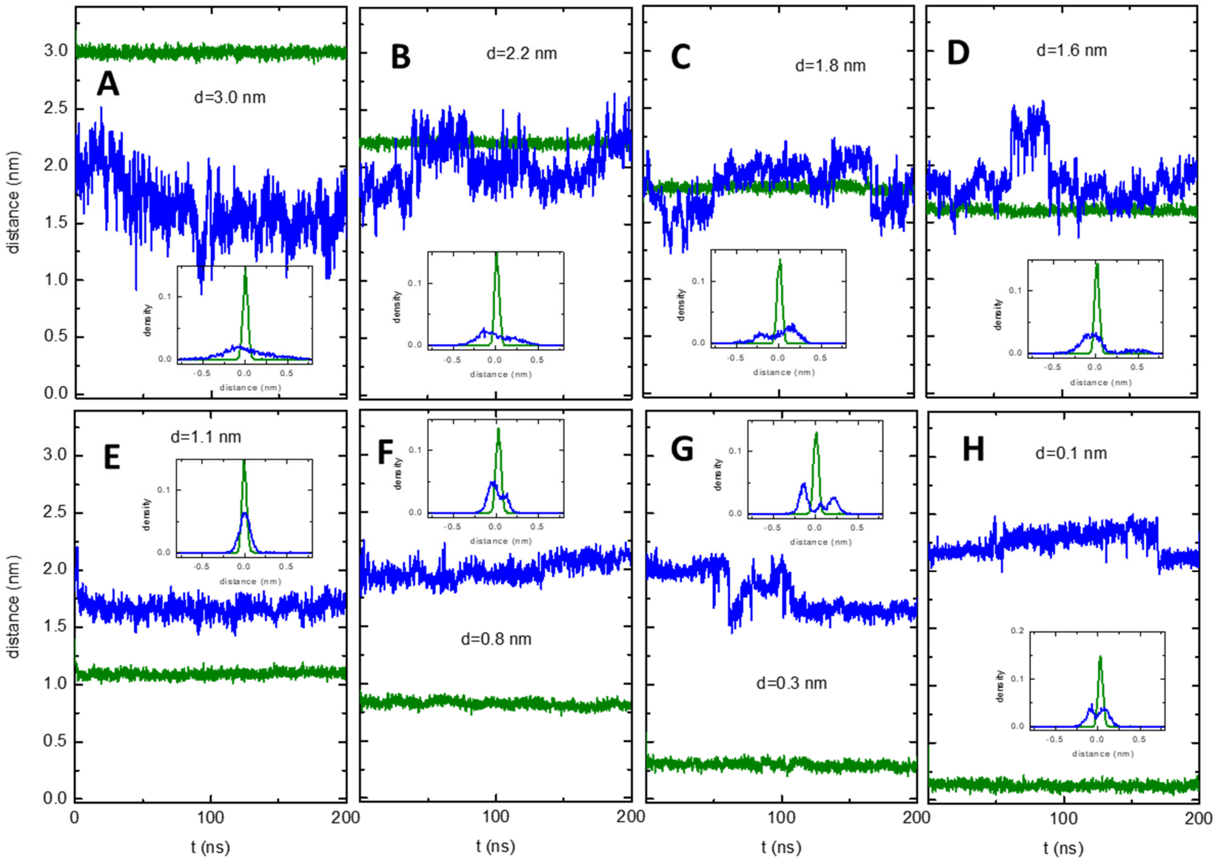

Figure S19 – Transverse location of the NBD group of NBD–C12 (*z*, —) and radial distance to the TM COM of P–gp (*d*, —) as a function of the simulation time in the windows corresponding to a restrained distance to the TM COM of P–gp (*d* = 3 nm (**A**), in the lipid bilayer; *d* = 2.2 and 1.8 nm (**B**,**C**), approaching the P–gp outer surface; *d* = 1.6 nm and 1.1 nm (**D**,**E**), near P–gp's entry gate; and *d* = 0.8, 0.3 and 0.1 nm (**F**,**G**,**H**), inside the P–gp binding pocket). The insets show the density of the NBD group around its average position in each window relative to the transverse (z, —) and to the constrained radial (*d*, —) distance.

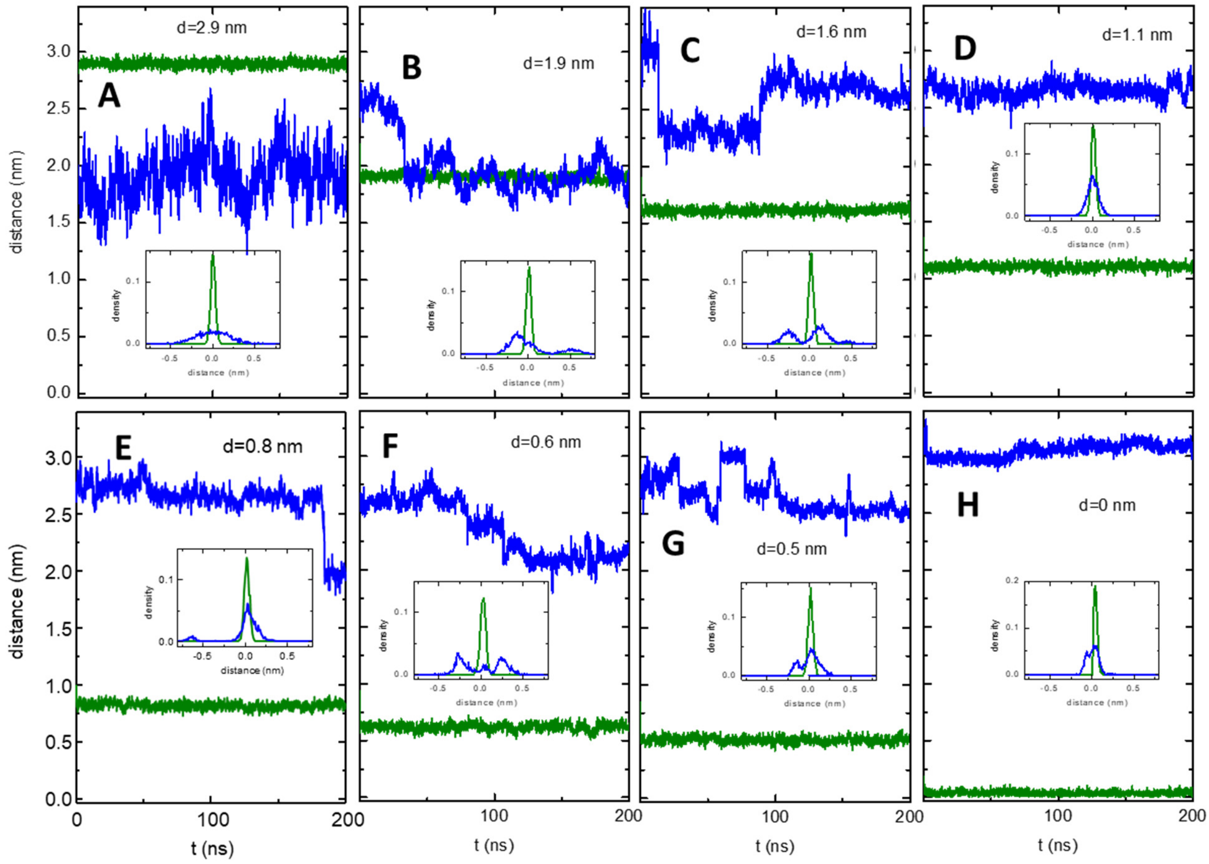

Figure S20 – Transverse location of the NBD group of NBD–C16 (*z*, —) and radial distance to the TM COM of P–gp (*d*, —) as a function of the simulation time in the windows corresponding to a restrained distance to the TM COM of P–gp (*d* = 2.9 nm (**A**), in the lipid bilayer; *d* = 1.9 nm (**B**), approaching the P–gp outer surface; *d* = 1.6 nm and 1.1 nm (**C**,**D**), near P–gp's entry gate; and *d* = 0.8, 0.6, 0.5 and 0 nm (**E**,**F**,**G**,**H**), inside the P–gp binding pocket). The insets show the density of the NBD group around its average position in each window relative to the transverse (z, —) and to the constrained radial (*d*, —) distance.

Table S3 – Summary of the interactions established between the NBD group of NBD-Cn and P-gp residues during the umbrella simulation for *d* = 0 nm. The residues that establish more contacts (above the 50th percentile) are highlighted in bold.

Molecule	P-gp region	Residues
NBD-C_4	TM3	Asp184, **Lys185**, Met188
	TM6	Gln343, Ala344, Ser345, **Asn347**
	TM10	**Met872**, **Leu875**, Ser876
	TM11	Phe938
	TM12	**Ser989**, **Phe990**, Ala991, Pro992, **Asp993**
NBD-C_8	TM3	Thr172, **Asp173**, Asp174, **Ser176**, **Lys177**, Glu180
	TM4	Thr236
	TM9	Thr812, Arg813, **Asn816**, Asp817, Gln820
	TM10	Lys883, Lys884
	TM12	Asp993, **Lys996**, Ser1000
NBD-C_{12}	TM3	Glu180, **Lys185**
	TM6	Gln343, Pro346, Asn347, Glu349
	TM10	Met872, **Leu875**, Ser876
	TM11	Phe938
	TM12	**Ser989**, **Phe990**, Ala991, **Pro992**, **Asp993**
NBD-C_{16}	TM3	**Asp173**, Asp174, **Ser176**, **Lys177**, Glu180
	TM9	Thr812, Arg813, **Asn816**, **Asp817**
	TM10	Lys884
	TM12	**Lys996**

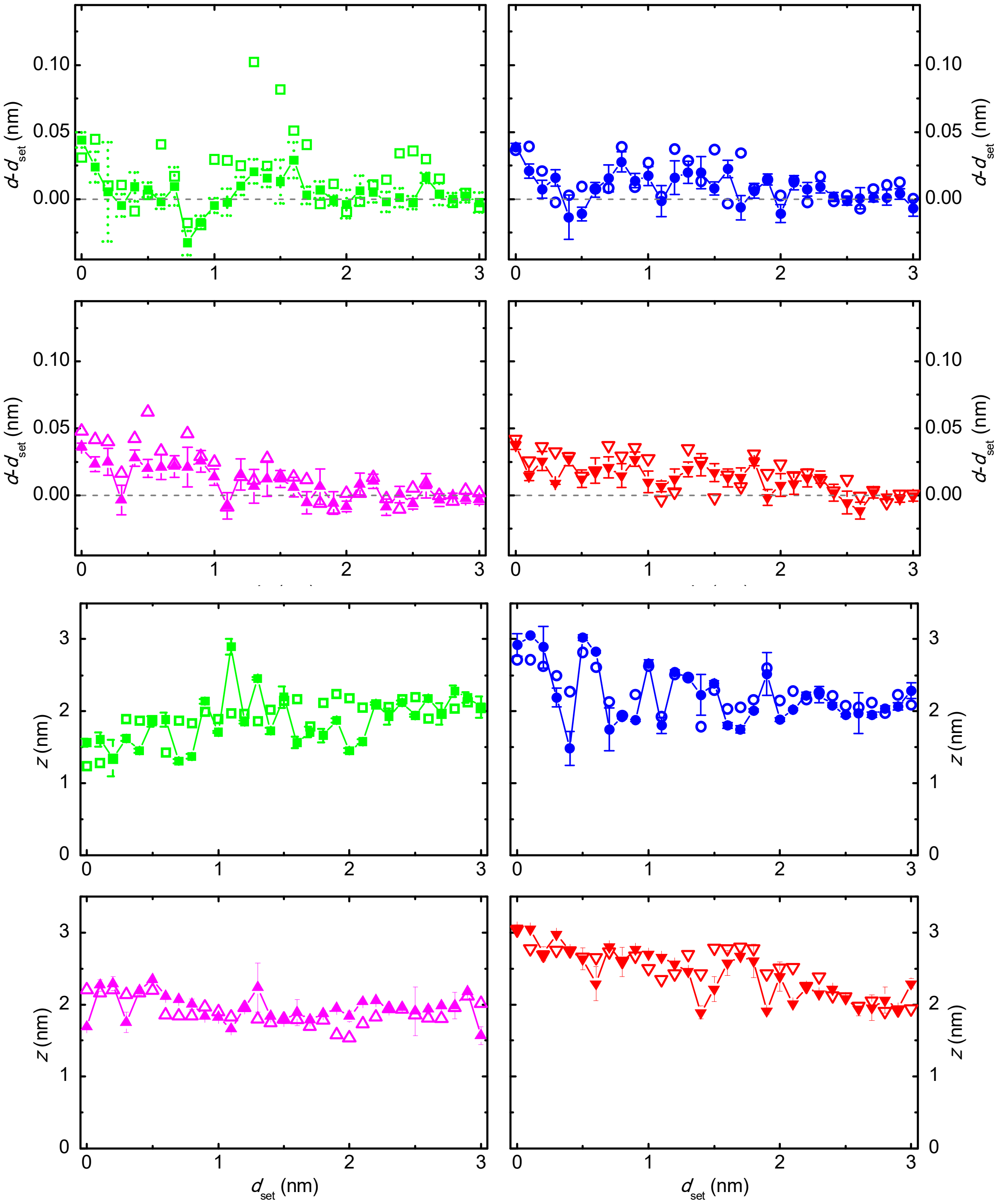

Figure S21 – Dependence of the initial (open symbols) and average over the 200 ns simulation (closed symbols) position of the NBD COM for NBD-C4 (□, ■), NBD-C8 (○, ●), NBD-C12 (▲, △), and NBD-C16 (▽, ▼) as a function of the restrained distance to the P-gp's TM COM (d_{set}). The upper plots show the distance to P-gp TM COM (d) and the lower plots the transverse location relative to the bilayer center (z).

NBDC4					
0 nm	0.5 nm (180 ns)	1 nm	2 nm (180 ns)	2.9 nm	5 nm
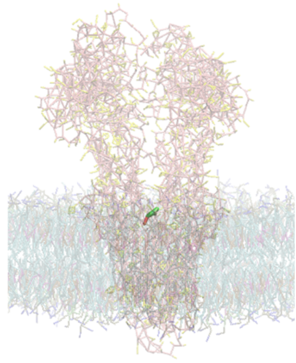	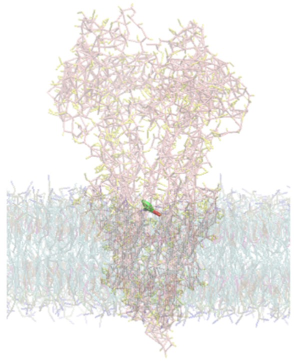	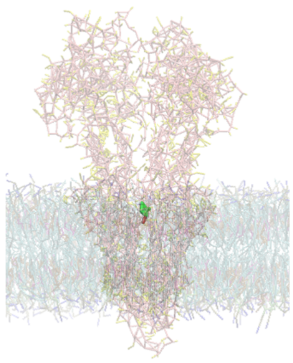	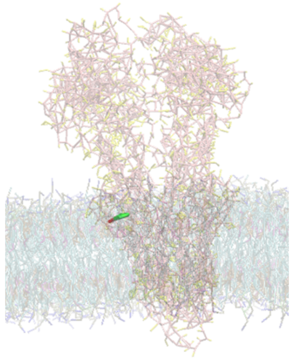	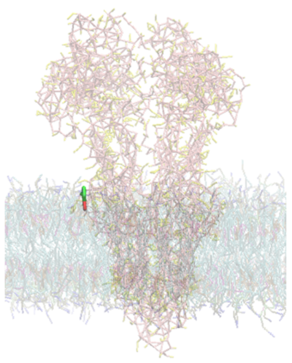	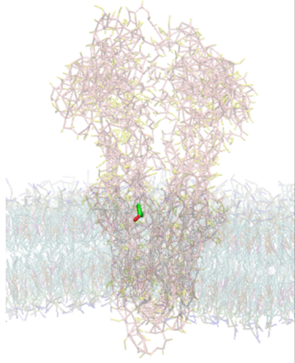
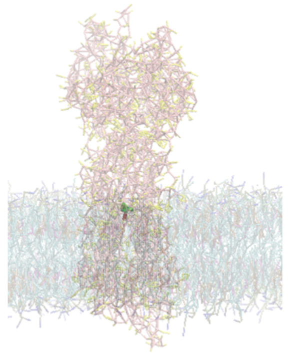	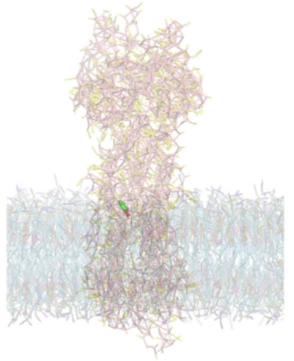	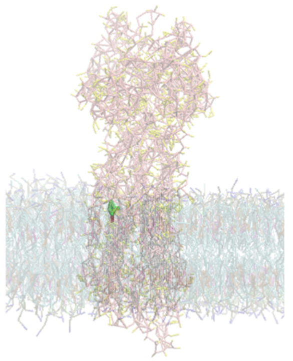	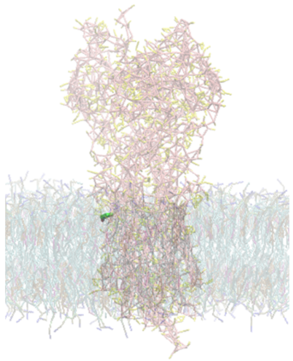	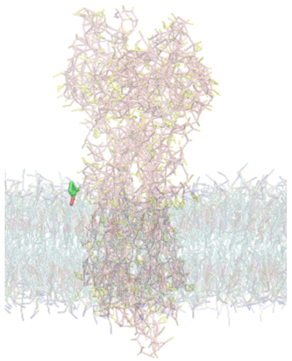	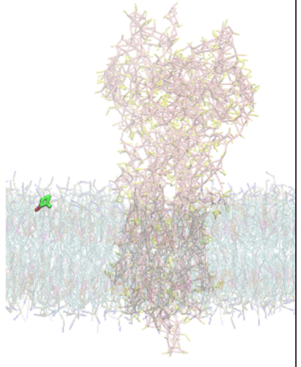

Figure S22 – Snapshots NBD-C4 with the NBD COM restricted at different distances from P-gp's TM COM, obtained at the end of the 200 ns MD simulation (or other representative time point, if specified). In top images the P-gp's gate between TM4/6 is facing to front, and in bottom images it is facing to the (left) side.

.

NBDC8					
0 nm (160 ns)	0.5 nm	1 nm (100 ns)	2 nm	2.9 nm	5 nm
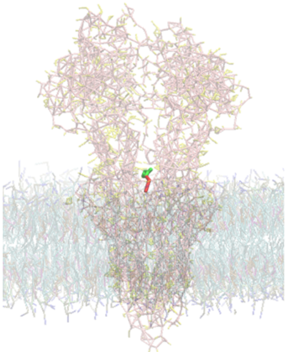	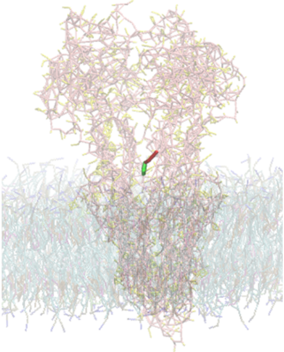	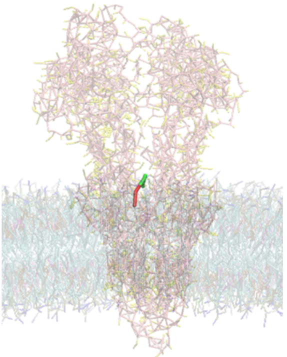	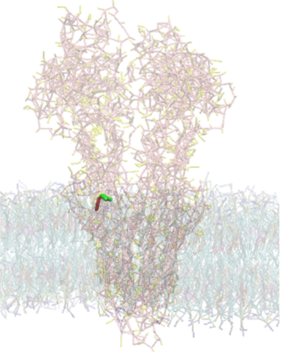	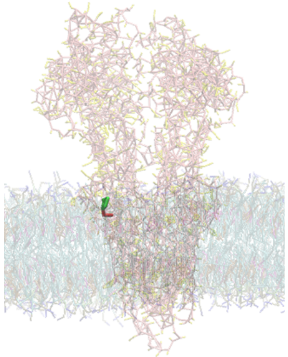	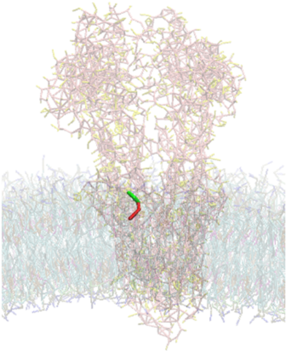
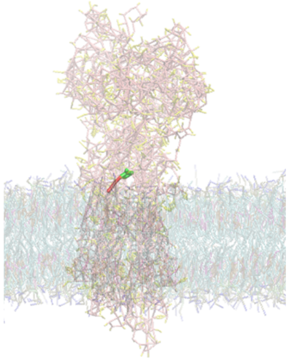	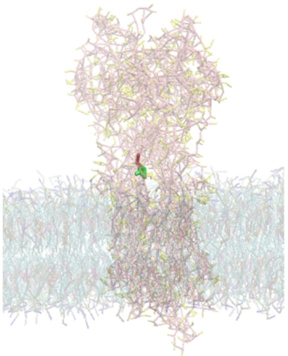	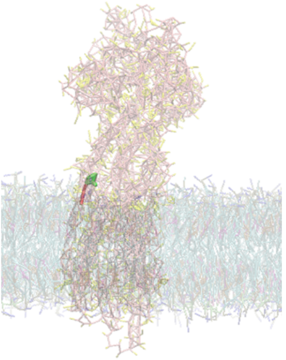	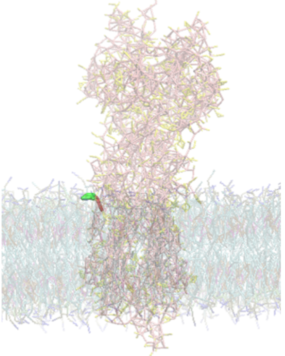	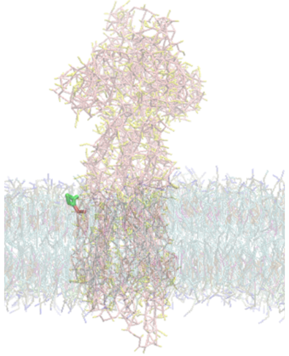	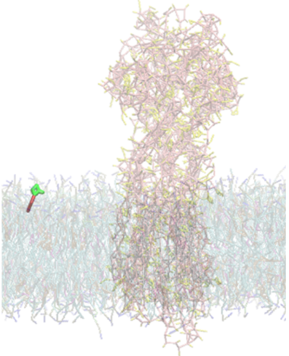

Figure S23 – Snapshots NBD-C8 with the NBD COM restricted at different distances from P-gp's TM COM, obtained at the end of the 200 ns MD simulation (or other representative time point, if specified). In top images the P-gp's gate between TM4/6 is facing to front, and in bottom images it is facing to the (left) side.

.

NBDC12					
0 nm	0.5 nm	1 nm	2 nm	2.9 nm	5 nm

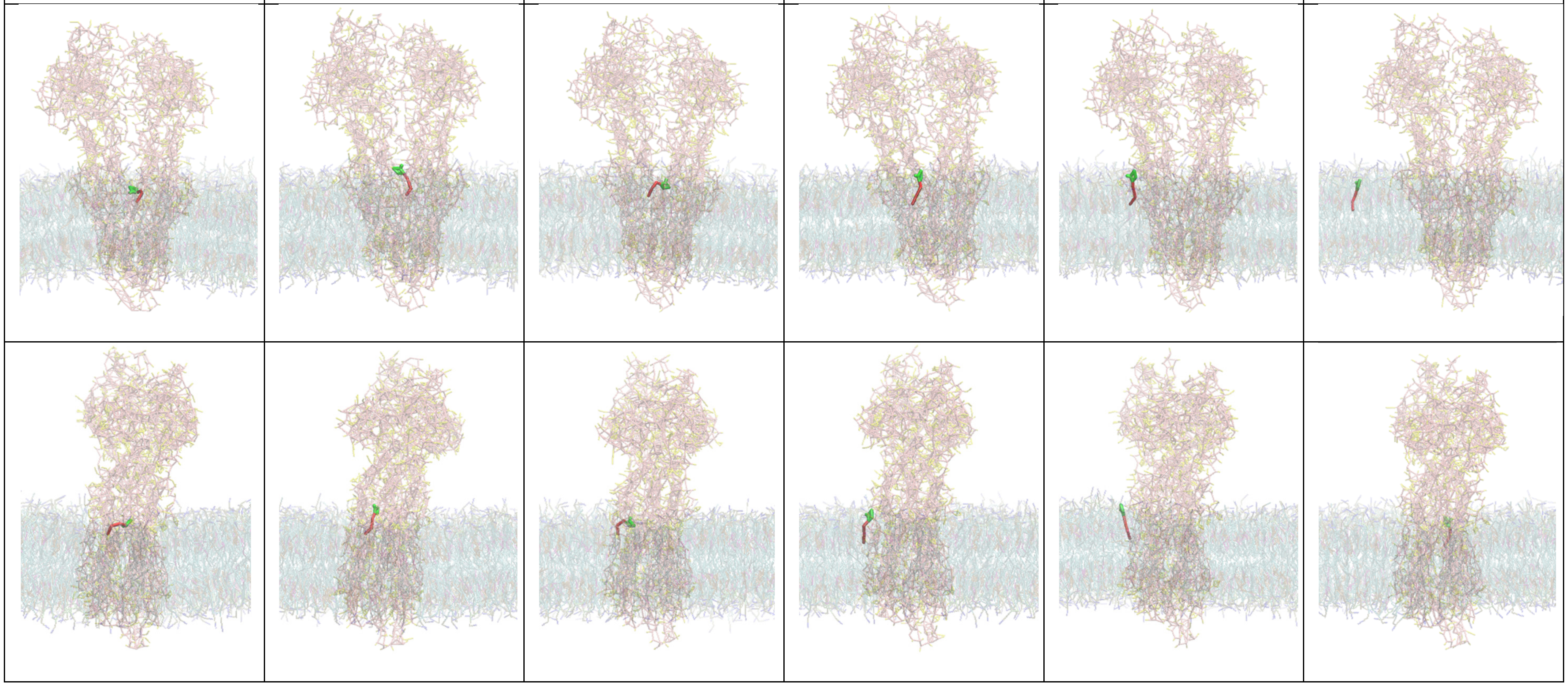

Figure S24 – Snapshots NBD-C12 with the NBD COM restricted at different distances from P-gp's TM COM, obtained at the end of the 200 ns MD simulation (or other representative time point, if specified). In top images the P-gp's gate between TM4/6 is facing to front, and in bottom images it is facing to the (left) side.

.

NBDC16					
0 nm	0.5 nm	1 nm (180 ns)	2 nm (160 ns)	2.9 nm	5 nm
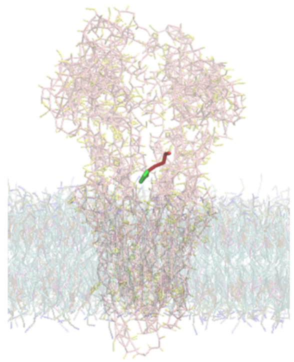	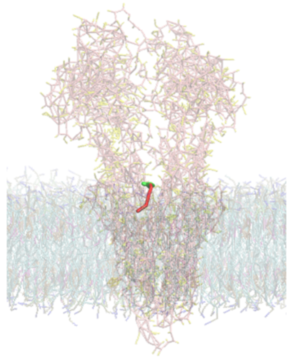	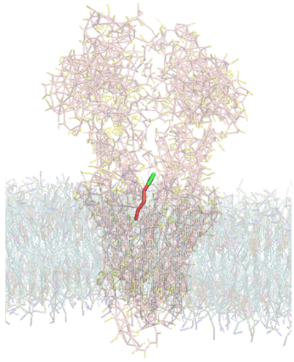	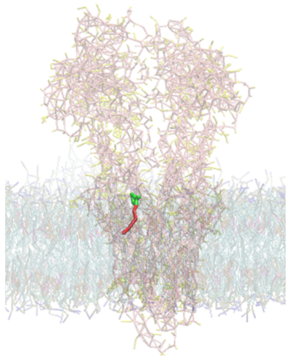	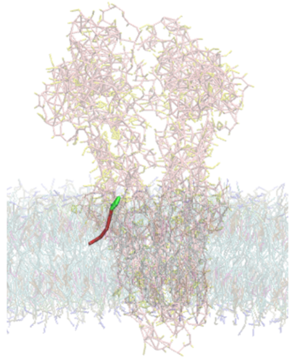	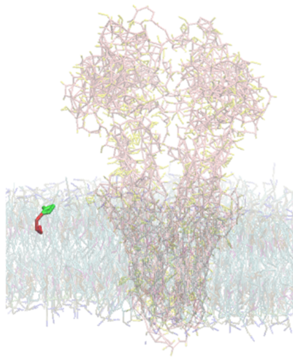
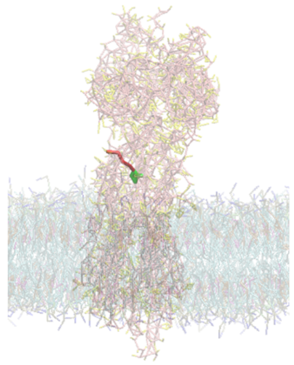	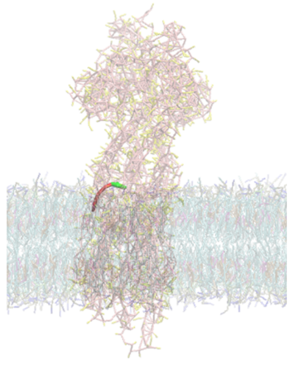	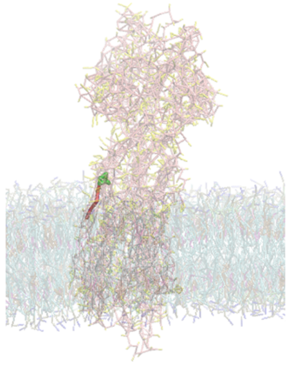	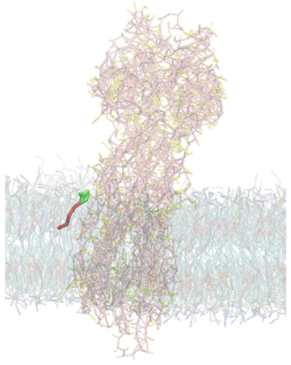	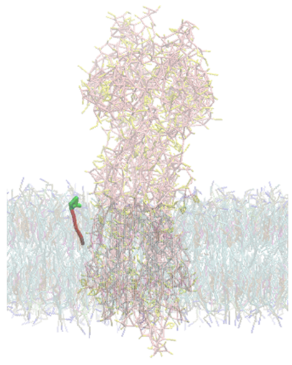	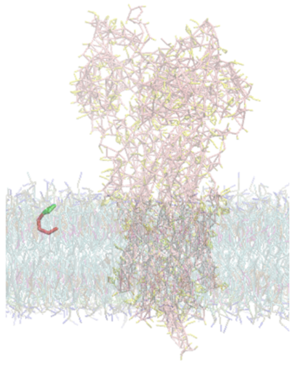

Figure S25 – Snapshots NBD-C16 with the NBD COM restricted at different distances from P-gp's TM COM, obtained at the end of the 200 ns MD simulation (or other representative time point, if specified). In top images the P-gp's gate between TM4/6 is facing to front, and in bottom images it is facing to the (left) side.

.

S10 – Calculation of the local concentration of the amphiphiles in the membrane and in the aqueous medium.

The concentration of phospholipids (PL) in a solution containing native membranes is calculated from the concentration of protein and the ratio of PLs to proteins (0.57 g PL/g P , average PL molar mass of 750 g/mol [1]). The volume of the lipid bilayer (V_{Lb}) is then calculated considering a density of 1 g/cm^3 for the phospholipids.

Typical values for the conditions of the ATPase activity assays are given below:

[P] = 0.107 mg/mL = 0.107 g/L;

[PL]=0.57 × 0.107 = 0.061 g/L = 0.061 g/750 g/mol) mol/L = 8.1×10^{-5} M

V_{Lb} = [PL] (g/L)× 1000 L/g = $6.1 \times 10^{-5} \times V_T$

The volume occupied by proteins in the membrane (V_P) was calculated considering a density of 1.2 g/cm^3 (being $8.9 \times 10^{-5} \times V_T$, and the total volume of native membranes is $1.5 \times 10^{-4} \times V_T$.

The IAAP displacement assay was performed with a protein concentration of 1 g/L, leading to proportionally higher volumes of the lipid bilayer and proteins.

Knowing the partition coefficients of the ligand one may calculate the fractions in each medium:

$$f_{\mathrm{L}}^{\mathrm{Lb}} = \frac{K_{\mathrm{P}}^{\mathrm{W}\to\mathrm{Lb}}\, V_{\mathrm{Lb}}}{V_{\mathrm{W}} + K_{\mathrm{P}}^{\mathrm{W}\to\mathrm{Lb}}\, V_{\mathrm{Lb}} + K_{\mathrm{P}}^{\mathrm{W}\to\mathrm{P}}\, V_{\mathrm{P}}}\,;\; f_{\mathrm{L}}^{\mathrm{P}} = \frac{K_{\mathrm{P}}^{\mathrm{W}\to\mathrm{P}}\, V_{\mathrm{P}}}{V_{\mathrm{W}} + K_{\mathrm{P}}^{\mathrm{W}\to\mathrm{Lb}}\, V_{\mathrm{Lb}} + K_{\mathrm{P}}^{\mathrm{W}\to\mathrm{P}}\, V_{\mathrm{P}}}$$

$$f_{\mathrm{L}}^{\mathrm{W}} = \frac{V_{\mathrm{W}}}{V_{\mathrm{W}} + K_{\mathrm{P}}^{\mathrm{W}\to\mathrm{Lb}}\, V_{\mathrm{Lb}} + K_{\mathrm{P}}^{\mathrm{W}\to\mathrm{P}}\, V_{\mathrm{P}}}\,.$$

The fraction of amphiphile in the membrane (lipid bilayer *plus* membrane proteins), is given by:

$$f_{\mathrm{L}}^{\mathrm{M}} = f_{\mathrm{L}}^{\mathrm{Lb}} + f_{\mathrm{L}}^{\mathrm{P}} = \frac{K_{\mathrm{P}}^{\mathrm{W}\to\mathrm{Lb}}\, V_{\mathrm{Lb}} + K_{\mathrm{P}}^{\mathrm{W}\to\mathrm{P}}\, V_{\mathrm{P}}}{V_{\mathrm{W}} + K_{\mathrm{P}}^{\mathrm{W}\to\mathrm{Lb}}\, V_{\mathrm{Lb}} + K_{\mathrm{P}}^{\mathrm{W}\to\mathrm{P}}\, V_{\mathrm{P}}} = \frac{K_{\mathrm{P}}^{\mathrm{W}\to\mathrm{M}}\, V_{\mathrm{M}}}{V_{\mathrm{W}} + K_{\mathrm{P}}^{\mathrm{W}\to\mathrm{Lb}}\, V_{\mathrm{Lb}} + K_{\mathrm{P}}^{\mathrm{W}\to\mathrm{P}}\, V_{\mathrm{P}}}\,,$$

where $K_{\mathrm{P}}^{\mathrm{W}\to\mathrm{M}}$ is the partition coefficient obtained experimentally between the aqueous media and the native membranes.

The above equations are only valid if the system is away from saturation (at low concentrations of amphiphiles relative to the binding agents). At relatively high amphiphile concentrations, the proteins may become saturated, leading to a decrease in the apparent partition coefficient and to an increase in the relative fraction of amphiphile associated with the lipid bilayer [2].

From the above equations one may calculate directly the concentration of amphiphile in each media:

$$\left[L_x\right] = \frac{\left[L_{\mathrm{T}}\right] f_{\mathrm{L}}^{\mathrm{x}}}{V_{\mathrm{T}}}$$

To evaluate if the aqueous medium is saturated, this concentration should be compared with the ligand critical aggregation concentration. Possible perturbation of the lipid bilayer is best evaluated from the molar ratio of lipids to ligand bound (PL:L)

$$PL : L = \left[PL\right]$$

S11 – Additional information for the re-analysis of the ATPase activity assay considering the local concentrations and several binding sites in P-gp's binding pocket.

Table S4 – Parameters for the best fit of the effect of NBD-C4 on P-gp's ATPase activity

n	K_d (μM)	β_1 (M-1)	V0 (%)	V1 (%)	V2 (%)	V3 (%)	V4 (%)	V5 (%)
1	75	1.3×10^4	90	703				
2	18	1.1×10^5	100	100	535			
3	11	2.8×10^5	100	109	109	512		
4	7	5.4×10^5	100	110	110	110	501	
5	9	5.5×10^5	100	110	110	110	259	491

Table S5 – Parameters for the best fit of the effect of NBD-C8 on P-gp's ATPase activity

n	K_d (μM)	β_1 (M-1)	V0 (%)	V1 (%)	V2 (%)	V3 (%)	V4 (%)	V5 (%)
1	3	3.0×10^5	100	286				
2	11	1.9×10^5	100	388	0			
3	4	7.8×10^5	100	112	403	65		
4	4	1.0×10^6	99	113	260	317	62	
5	6	8.6×10^5	100	122	275	253	253	0

Table S6 – Parameters for the best fit of the effect of NBD-LysoMPE on P-gp's ATPase activity

n	K_d (μM)	β_1 (M-1)	V0 (%)	V1 (%)	V2 (%)	V3 (%)	V4 (%)	V5 (%)
1	20	5.0×10^4	105	0				
2	6	3.1×10^5	100	122	0			
3	3	9.2×10^5	99	99	146	0		
4	3	1.4×10^6	100	100	100	129	0	
5	3	1.7×10^6	100	100	100	116	97	0

S12 – Additional information for the re-analysis of the IAAP displacement assay considering the local concentrations and several binding sites in P-gp's binding pocket.

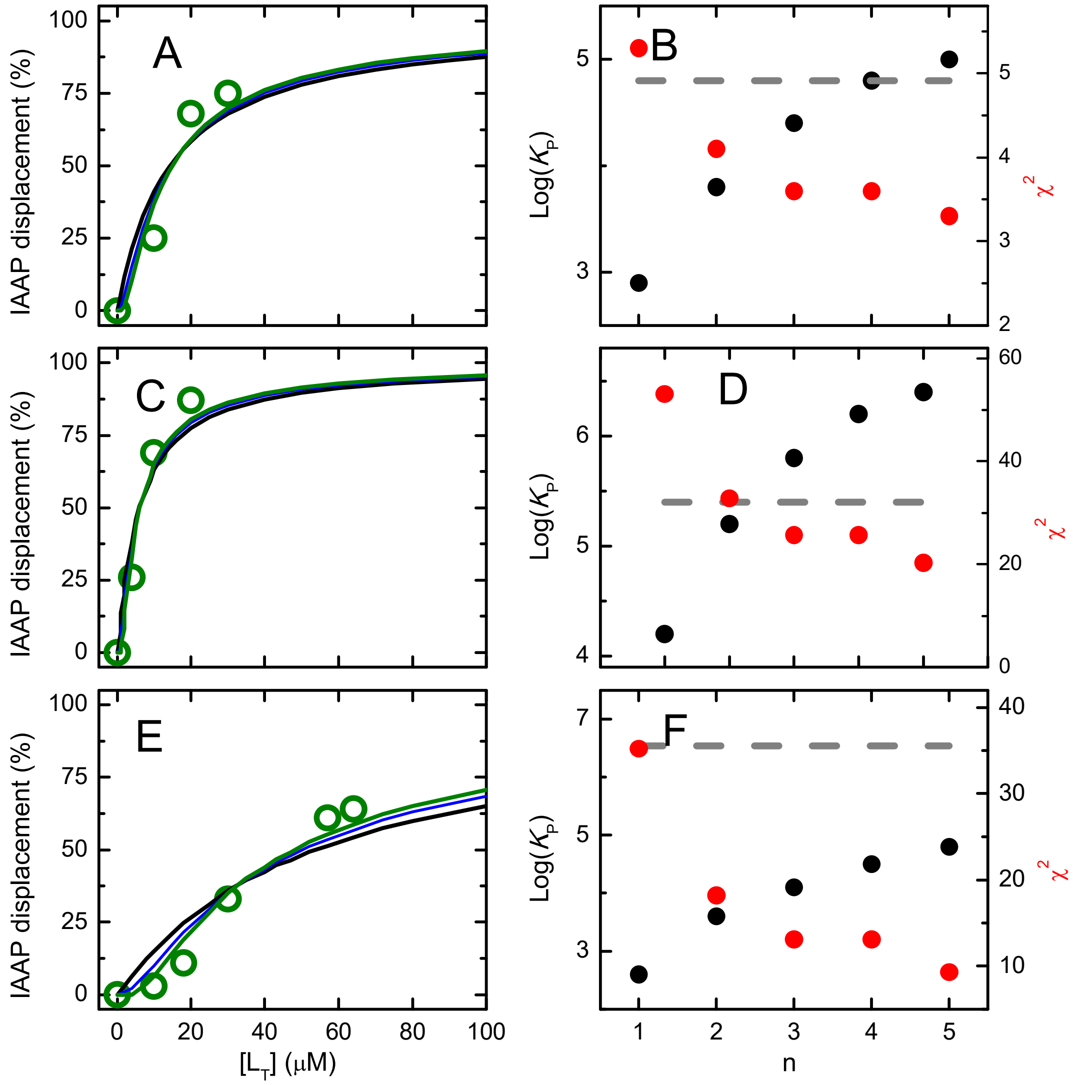

Figure S26 – Effect of NBD-C4 (plot A and B), NBD-C8 (plot C and D), and NBD-LysoMPE (plot E and F) on IAAP displacement from P-gp's binding pocket considering that IAAP is displaced from P-gp's binding pocket only when all binding sites are occupied by the NBD amphiphile (i=n). The plots on the left show the experimental results (○) and the best fit of equation (11) from the manuscript, considering different number of binding sites in P-gp's binding pocket: n =1 (—), n =2 (—), and n =5 (—). The plots on the right show the effect of the number of binding sites on the quality of the best fit (χ^2, ●), and on the partition coefficient calculated from β_1 (●). The partition coefficient between the aqueous phase and P-gp obtained from the partition experiments is also shown (- - -).

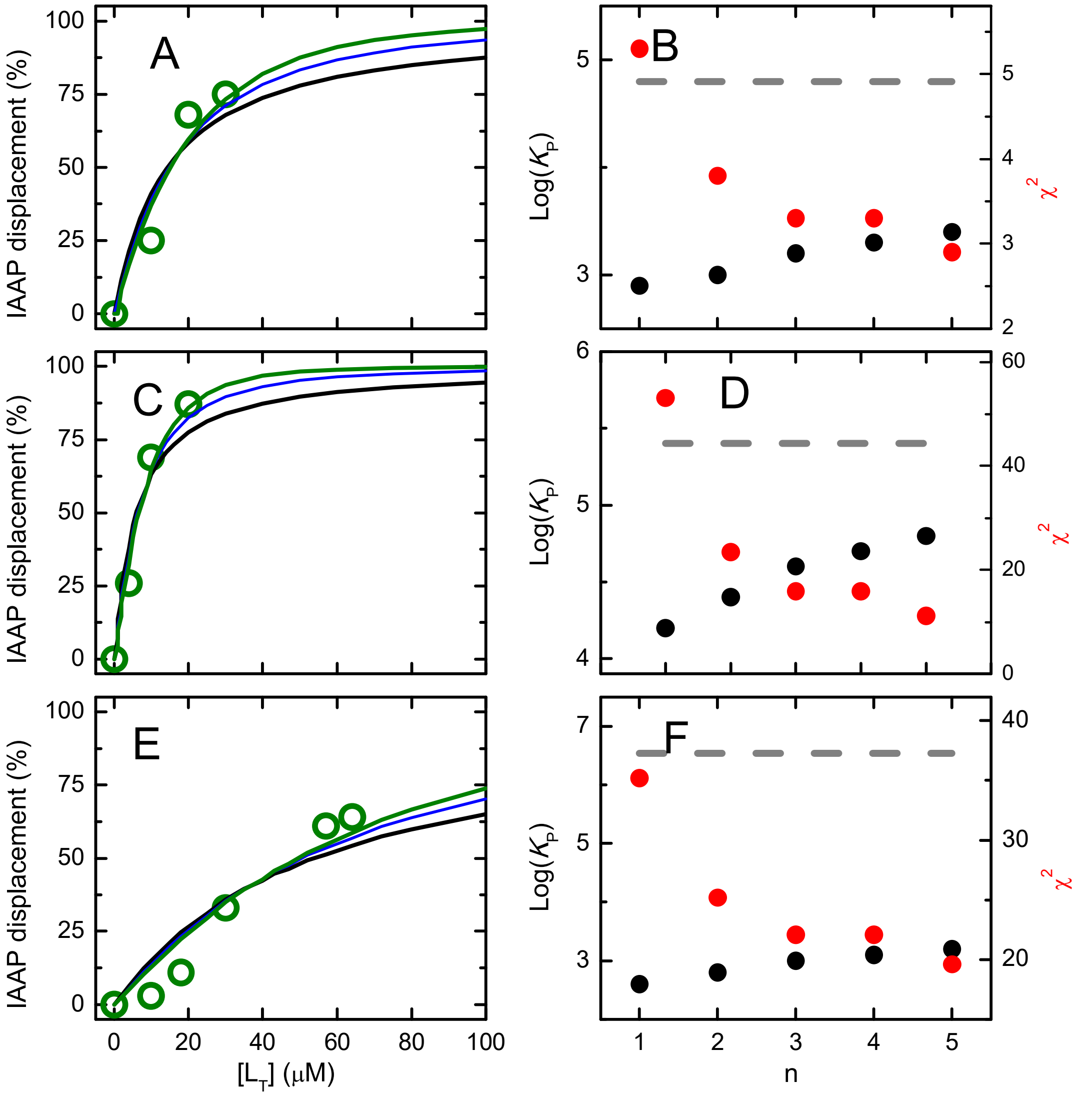

Figure S27 – Effect of NBD-C4 (plot A and B), NBD-C8 (plot C and D), and NBD-LysoMPE (plot E and F) on IAAP displacement from P-gp's binding pocket considering that IAAP is displaced from P-gp's binding pocket when at least one NBD amphiphile is bound (i≥1). The plots on the left show the experimental results (○) and the best fit of equation (11) from the manuscript, considering different number of binding sites in P-gp's binding pocket: n =1 (—), n =2 (—), and n =5 (—). The plots on the right show the effect of the number of binding sites on the quality of the best fit (χ^2, ●), and on the partition coefficient calculated from β_1 (●). The partition coefficient between the aqueous phase and P-gp obtained from the partition experiments is also shown (- - -).

References

1. Moreno, M.J.; Martins, P.A.T.; Bernardino, E.F.; Abel, B.; Ambudkar, S.V. Characterization of the Lipidome and Biophysical Properties of Membranes from High Five Insect Cells Expressing Mouse P-Glycoprotein. *Biomolecules* **2021**, 11, 426, doi:10.3390/biom11030426.
2. Moreno, M. J.; Loura, L. M. S.; Martins, J.; Salvador, A.; Velazquez-Campoy, A., Analysis of the Equilibrium Distribution of Ligands in Heterogeneous Media - Approaches and Pitfalls. *International Journal of Molecular Sciences* **2022**, 23, 9757, doi: doi:10.3390/ijms23179757.
